# Exploring health providers’ and community perceptions and experiences with malaria tests in South-East Nigeria: a critical step towards appropriate treatment

**DOI:** 10.1186/1475-2875-11-368

**Published:** 2012-11-06

**Authors:** Ogochukwu P Ezeoke, Nkoli N Ezumah, Clare CI Chandler, Lindsay J Mangham-Jefferies, Obinna E Onwujekwe, Virginia Wiseman, Benjamin S Uzochukwu

**Affiliations:** 1 Health Policy Research Group, Department of Pharmacology and Therapeutics, College of Medicine, University of Nigeria, Enugu, Nigeria; 2 Department of Sociology/Anthropology, University of Nigeria, Nsukka, Nigeria; 3 Department of Global Health and Development, London School of Hygiene and Tropical Medicine, London, UK; 4 Department of Health Administration and Management, University of Nigeria, Enugu Campus, Nsukka, Nigeria; 5 Department of Community Medicine, College of Medicine, University of Nigeria, Enugu Campus, Nsukka, Nigeria

## Abstract

**Background:**

The adoption of ACT as the first line treatment for uncomplicated malaria in Nigeria has concentrated attention on the role of testing in appropriate malaria treatment. There are calls at both national and global level for malaria treatment to be based on test result, but it is still unclear how testing can be incorporated into treatment-seeking and practices of health providers. This study explored community members and health providers’ perceptions and experiences with malaria tests in south east Nigeria.

**Methods:**

The study was conducted in urban and rural areas of Enugu state in south-eastern Nigeria. A total of 18 focus group discussions with 179 community members including sub-groups of primary caregivers, adult men and adult women aged 15 years and above. Twenty- six (26) In-depth interviews were held with public and private health providers involved in prescribing medicines at public and private health facilities in the study area.

**Results:**

Both providers and community members were familiar with malaria tests and identified malaria tests as an important step to distinguish malaria from other illnesses with similar symptoms and as a means of delivering appropriate treatment. However, the logic of test-directed treatment was undermined by cost of test and a lack of testing facilities but above all concerns over the reliability of negative test results, with community members and providers observing inconsistencies between results and symptoms, and providers attributing inaccurate results to incompetencies of technicians. Recognition of malaria symptoms was deemed most important in determining the use of antimalarial drugs rather than the result of a malaria test.

**Conclusion:**

The results highlight important areas of intervention to promote appropriate malaria treatment. If tests are to play a role in patient management, demand and supply side interventions are needed to change people’s attitude towards malaria test results.

## Background

The adoption of artemisinin combination therapy (ACT) as first line treatment for malaria by many African countries including Nigeria has increased the emphasis on accurate malaria diagnosis before treatment to prevent the indiscriminate use of these drugs. In Nigeria, the policy to treat malaria with ACT specifies that treatment should be based on a parasitological test result where testing is available [1]. There has been a global call for parasitological confirmation by microscopy or with a rapid diagnostic test (RDT) for patients of all ages with suspected malaria [2]. The Roll Back Malaria partnership also set new targets of universal access to malaria diagnostic testing in public and private sectors as well as at the community level [3], but evidence suggests limited role of tests in Nigeria for malaria case management with only about 5% of children under-5 with fever being tested [4]. The scaling-up of diagnostic testing is critical to ensure appropriate management of malaria and non-malarial febrile illnesses and target anti-malarial medicines to those who actually need them [3]. In Nigeria it is not clear how these tests are perceived and how they could be incorporated into current treatment-seeking and prescribing practices of health care providers.

Studies have shown that reliable diagnostic testing is severely limited in Africa, and misdiagnosis commonly occurs, which exacerbates morbidity, promotes the perception that testing is not useful, and compromises patient care [5]. Where these tests are being provided, there are common perceptions and beliefs about their accuracy and impact on patient care. For instance in Tanzania, a study found that although clinicians and patients/caretakers were comfortable with overall performance of laboratories, test results had limited impact on patient management [6]. In Nigeria, a study on availability and use of RDTs in public and private health facilities found limited use of RDTs in these facilities the main reasons given for non-use were unreliability of RDTs, supply issues, and preference for other methods of diagnosis [7].

In a number of settings, the introduction of RDTs has not been found to affect case management, with many patients continuing to be treated for malaria with negative results [8,9]. Social research across different settings has suggested that this over diagnosis is part of the culture of care in areas with a long history of malaria and that simply introducing new technologies to identify parasites fits poorly with priorities of local providers and patients [10,11]. Concerns over testing also arise from studies that have described how the practice of taking blood may be interpreted differently to biomedical intentions [12,13]. For instance, in Uganda, it was reported how community members feared that blood collected for malaria testing could be used for HIV testing or witchcraft and that children could be infected with HIV leading the authors to suggest that a well-designed behaviour change communication strategy is needed to address the anticipated programmatic challenges [14].

The Government of Nigeria plans to introduce RDTs in health facilities in Nigeria, but little is known from the social perspective about what challenges may be faced with implementation and scale up of these tests in this setting. Since the acceptance of any healthcare intervention depends on people’s perception of the innovation amongst other factors [15], it becomes crucial to explore people’s perception and experiences with malaria tests. Prior to a randomized control trial where RDTs will be introduced to health care providers and community members educated on the importance of appropriate diagnosis and treatment, the views of providers and community members who are potential recipients of these interventions were explored. This paper presents findings from this baseline study undertaken in south-eastern Nigeria.

## Methods

### Study area

The study was carried out in Enugu state in 2010. The state is geographically located in the south-eastern zone of Nigeria. It has a land area of 7,700 square kilometres and a population of over 3 million people [16]. Two sites were chosen; Enugu which is the largest predominantly urban area in Enugu State and Udi a rural area. The study sites are similar in culture and have a mix of both public and private facilities. Malaria is endemic in both sites, and occurs all year round. Government provides health care services through the primary health centres, secondary and tertiary hospitals. There are in addition, private laboratories and hospitals together with pharmacies and patent medicine dealers (PMDs) that are privately owned. At primary health facilities, treatment of malaria is free for pregnant women and children under-5 despite, although studies show that pharmacies and PMDs have remained a major source for the treatment of malaria [17]. Pharmacies and PMDs are licensed to sell over the counter pharmaceutical products. These facilities are medicine retailers and do not routinely offer clinical care or diagnostic services. A few primary health care facilities offer diagnostic tests [9].

### Study design

Information was collected through qualitative methods which consisted of focus group discussions (FGDs) with community members and in-depth interviews (IDIs) with health care providers in the two study areas. In each of the site, three (3) communities were randomly selected and in each community FGDs were held with three sub-groups of community members chosen to represent those seeking care most frequently: primary caregivers, adult men and adult women aged 15 years and above. Women of 15 years and above were included because at this age, they are considered old enough to marry. A total of 18 focus group discussions were conducted with 179 community members.

Health facilities were selected from a list obtained from census of health facilities (both public and private) in the study area. A total of 26 health facilities were included in the study, 13 facilities in each site. In all, twenty- six (26) in-depth interviews were conducted with public and private health providers including nurses, midwives, nursing assistants, community Health Extension Workers, Community Health Officers, Pharmacy Representatives and patent medicine dealers involved in prescribing medicines.

### Selection of participants and data collection

FGDs were held with community members, prior to the interviews, the research team identified members of the communities as contact persons. Selection of participants was carried out by approaching contact persons for the research in each of the study communities and asking them to invite 8-12 participants according to the inclusion criteria at a certain date and time to a convenient venue. Topics for the FGDs included knowledge of and experience with malaria test, preferences and trust in test results, views about importance of tests and whether they will be happy to go for tests if made available.

In-depth interviews (IDIs) were held with health care providers from selected public and private facilities. This method was chosen for providers because of their heterogeneity in cadre and geographical distances. The facilities included were primary health centres, patent medicine dealers and pharmacies. These types of facilities were chosen because they serve as the commonest source of malaria treatment in this region. In each of the selected health facilities, one health provider was interviewed who has a role in prescribing or dispensing. Topics for the in-depth interviews included role of malaria diagnosis and testing in their treatment and prescribing practices, views and experience with diagnostic tests, trusts and logistics of diagnostic tests and views on community preferences for tests.

For both FGDs and IDIs, demographic information of participants including age, occupation, marital status, level of education was recorded using the enrolment forms. At the start of the interviews, participants were given information sheets and told the purpose of the study. They were then asked to provide written consent. Where written consent could not be provided, oral consent was obtained. Participants were given codes to ensure anonymity and interviews were coded according to study site and participant category. The study team comprised of seven facilitators and seven note-takers trained over 5 days using a standard social science field manual developed for the study. Interviews commenced after the pre-test and revision of study tools. Two (2) coordinators and a lead sociologist supervised the interviews. The facilitators moderated the interviews following a topic guide. Responses were recorded with digital voice recorders as well as through hand-written notes; an observer was present at each interview to ensure that all the questions were covered. All FGDs and IDIs were conducted in the native Igbo language.

### Data management

The audio files of FGDs and IDIs were transferred to the computer immediately after each interview and were transcribed and translated by trained note takers within 48 hours of conducting the interview. All interviews were proof-read by the supervisors to ensure the accuracy and consistency of transcription. Each interview was labelled according to site and category of participant(s).

### Data analysis

Transcripts and information from enrolment forms were imported into NVivo software version 8 (QSR International). They were read carefully and coded according to the responses of the respondents. The first three transcripts were done by three different people and then merged; this was done to ensure agreement of a coding template between the coders. Emerging ideas were identified and grouped into themes in a continuous revision process as more transcripts were reviewed. Two sociologists then completed the coding. Analysis involved drawing together themes based on the questions asked.

### Ethical aspects

Approval to carry out the study was obtained from the Ethics Review Board of the University of Nigeria (UNTH/CSA.329) and the London School of Hygiene & Tropical Medicine (Approval 5429).

### Findings

A total of 179 community members participated in the FGD and in more than half of the FGD groups, the number of participants ranged from 10-12 (Table 1). The lowest mean age of participant groups was 26 and the highest was 55. Participants were all married and in about half of the groups, the highest level of education was secondary. In only a few groups were most people unemployed. Table 2 shows that 58% of IDI respondents were private drug retailers. The highest level of education for majority was junior secondary education. The mean age of respondents was 38 years. Majority of the respondents were not originally from the area and most (54%) have worked for about 1-5 years in the facilities. The highest number of years of professional training for the majority ranged from 11-15 years.

**Table 1:** Demographic details of FGD participants

**FGD GROUP (IDNO)**	**no of participants**	**Age [mean]**	**Status in the family [mode]**	**Marital status [mode]**	**Highest level of education [mode]**	**occupation [mode]**	**Number of children [mean]**
FUNAAM01	11	54.9	Head	Married	Primary	Farmer	2
FUNAPC01	9	26.1	Wife	Married	Secondary	Unemployed	2
FUNSAM01	10	49.6	Head	Married	Primary	Farmer	4
FUNSAW01	8	49.6	Wife	Married	Primary	Farmer	5
FUNSPC01	9	26.1	Wife	Married	Secondary	Unemployed	2
FUNSAM01	9	49.6	Head	Married	Primary	Farmer	4
FUUMAW01	11	45	Wife	Married	Uneducated	Farmer	5
FUUMPC01	8	27	Wife	Married	Secondary	Trader	1
FUNAAW01	12	31.1	Wife	Married	Secondary	Trader	4
FEABAM01	11	47	Head	Married	Primary	Artisan	4
FEABAW01	12	34.6	Wife	Married	Secondary	Trader	4
FEABPC01	9	29.3	Wife	Married	Secondary	Unemployed	3
FENHAM01	12	53.17	Head	Married	Primary	Trader	5
FENHAW01	9	28.6	Wife	Married	Secondary	Civil Servant	3
FENHPC01	10	32.6	Wife	Married	Secondary	Trader	4
FEOGAM01	10	43.3	Head	Married	Primary	Civil Servant	1
FEOGAW01	12	27.8	Wife	Married	Secondary	Artisan	3
FEOGPC01	7	32.1	Wife	Married	Secondary	Civil Servant	3

**Table 2:** Demographic details of IDI participants

**Variable**	**N = 26n(%)**
**Facility type**	
Public	11(42)
Private	15(58)
**Cadre of health worker**	
Drug retailer/pharmacist	15(57)
Nurse	2(8)
Nursing assistant, CHEWS, Other cadres	9(35)
**Age**	
30 and less	6(23)
31-41	8(31)
42-51	8(31)
52 and above	4(15)
**Highest level of education**	
Primary	2(8)
Junior secondary	8(31)
Senior secondary	0(0)
School of Health	7(27)
Tertiary	7(27)
Refuse to answer	2(8)
**Originally from region**	
Yes	8(31)
**Years worked at facility**	
<1-5	14(54)
6-10	6(23)
11- 15	1(4)
16 and above	5(18)
**Number of years of professional training**	
None	3(11)
<1 yr -5 yrs	10(38)
6 yrs-10 yrs	6(23)
11 yrs- 15 yrs	2(8)
16 yrs and above	5(18)

Key themes that were important across the different sub-groups of participants were identified which includes the frequency with which testing is used, the perceived importance of testing, reasons for not using malaria tests, trust in test results and when tests are undertaken how the test results are used. These issues are discussed further below.

### Frequency of testing

Participants in about half of the FGDs acknowledged having had malaria test. While they would not always subject themselves to tests at all times, they would usually take their children to be tested when they suspect them of having malaria.

"‘…… What I do is to buy drugs from chemist and my malaria is cured. But whenever my children become sick I immediately take them to the hospital, the first thing they do is to test them. And they will receive treatment’. (P3 FGD, Enugu, Adult Men)"

On the other hand, the perspective of the health care providers was that only a few people, would consent to testing; majority would typically ask to be treated straight away. Amongst the reasons given by health providers on why people do not demand tests was their perceived self-recognition of malaria and its symptoms.

"‘They feel they really know what is wrong with them. At times some will come and tell you that they have malaria so you should give them medicine for malaria, so when you try to convince them that it is necessary to undergo test so that the sickness will be well identified and positively treated, being villagers ignorantly they wouldn’t like to go for test’. (IDI, Udi, Drug Retailer)"

"‘Well for the period I have stayed with them here, just a few would like to go for test. And the few, I mean the elite among them’. (IDI, Udi, Drug Retailer)"

The providers also emphasized that the duration of the symptoms felt by patients affect whether they will be willing to go for tests or not since a prolonged fever is usually perceived as more serious

"‘If someone comes in here with fresh malaria that is, the one that started yesterday night or this morning and he comes in here and you start suggesting test He will not be willing but if one has had malaria for sometime let’s say last week, or last month and give this kind of story. We will say why don’t you go to the lab so that we will make sure it is not malaria? then that is the time he/she will submit and go for test’. (IDI, Enugu, Pharmacy)"

### Importance of testing: provider & community perspective

Across 11 FGDs, Participants emphasized, the importance of tests both for adults and children, in identifying the sickness that one has. In their explanations, treatment based on self-recognition of symptoms and clinical judgement may lead to missing out on treating the particular ailment one has as malaria symptoms overlap with those of other illnesses.

"‘A times you might be suspecting that what is wrong with you is malaria but when the test results comes out it will show that it is typhoid’. (P4 FGD Enugu, Adult Women)."

"‘…some people may not go for test and take the wrong drugs but if you go for test, they know that it is malaria and treat it directly’. (P7 FGD, Enugu, Adult Men)"

"‘I am advising all mothers, if your child is sick and you go to the hospital, tell the medical doctor to refer you people for test because it is test that will show the main thing that is wrong with your child. A times we mothers when our child is ill we start giving the child malaria drugs but when we then go for test, it will show us that what is wrong with our child is not malaria but typhoid’. (P6 FGD, Enugu, primary care giver)"

In the same manner, health providers’ (In 15 of the 26 IDIs) in both public and private facilities while outlining the ill effects of clinical diagnosis to patients described tests as desirable and very important in distinguishing malaria from other illnesses and to give appropriate treatment.

"‘It is very important that anybody coming to complain to non-medical or even a medical person,…it is advisable to go for test so that …. it won’t be a matter of guess work, because by so doing you may complicate the persons sickness, but if you advise or tell the person to do test in a very good lab, that will help to at least narrow down the chances of making mistakes giving somebody what the person might not require’. (IDI, Enugu, Drug Retailer)"

"‘It is very important. If we were doing it, we would have known if a patient has malaria actually or not instead of doing guess work. A patient might be suffering from typhoid and we will be treating malaria’. (IDI, Udi, Health Centre, Community Health Officer);"

### Importance of testing: categories of people providers send for test

Although testing was widely acknowledged to be important, it appeared that not everyone who presents with fever will usually be referred for a test. The health providers identified the different categories of patients they would normally refer for test. These quotes reflect the responses from the public providers:

"‘Ok like a pregnant woman, I will quickly tell her to go to the hospital and see a doctor or go for a lab check-up’. (IDI, Enugu, Community Health Officer)"

"‘Yes there are like some patients that will come they would name all the necessary malaria drugs and that they had taken at home and yet they are still having the symptoms you will now have to send them for test to really confirm what is the problem before you now administer your own treatment’. (IDI, Enugu, Health Centre, Nurse)"

"‘There are some patients that will come with some confusing signs and symptoms. They will present with so many signs and symptoms at the same time and you will not really be sure it is malaria then you have to send them for test’. (IDI, Enugu, Health Centre, Nurse)."

The private providers however were of the opinion that they will recommend tests to patients based on their perceived seriousness of the illness and the patient’s willingness to accept to be tested.

"‘Village mentality cannot be over emphasized. There are people you will refer to lab and from there they will go to another place and buy what they want to buy. So when you sit down with the person, dialogue with the person, if the person is understandable, not a group of person or particular types of person but people that will understand me, then I give them that referral’. (IDI Udi, Drug retailer)"

"‘Depending on the seriousness of the sickness, I might ask some to go for test, while I use my initiative for the others’. (IDI, Enugu, Drug Retailer)"

### Reasons for not testing: patients need money

When asked about reasons that people do not go for test, providers were generally of the opinion that financial constraints deter people from having a malaria test, in addition to cost issues an individual’s level of awareness also play a role:

"‘In my own understanding it is the issue of money. Anything that concerns money here is a different matter. If you tell the person, his response will be which money would I use for test? The person prefers to buy drugs given to him’. (IDI, Udi, Drug Retailer)"

"‘You see … very many of them do not have money they are peasants they would prefer that you treat malaria. But the few enlightened ones will say nurse I want to go to test then you give them the lab form for the test’. (IDI, Enugu, Nurse)"

"‘Yes they always say it is a waste of money for them to go for test. They prefer to be treated then if the sickness,… if they are not responding to the treatment being given to them they will now agree to go for test’. (IDI, Health Centre Nurse)"

### Reasons for not testing: lack of facilities for testing

Generally, for the health care providers in both public and private facilities, there were reports that they still treat based on signs and symptoms due to lack of equipments for testing.

"‘It is very important to test, because you can be treating for malaria, and it turns out to be something else, that is disturbing the person. But if you test you are sure of what you are doing. But in our own case, since we don’t have what we can use to test or a laboratory, we just be treating them like that and continue begging God’. (IDI, Udi, Community Health Officer)"

"‘It is very important but we don’t have the facility for testing’. (IDI, Enugu, Health Centre, Nurse)"

They also expressed the importance of having such facilities to enable them to give appropriate treatment as is currently being advocated by the National malaria control programme.

"‘Well, there are ….our impediments often time is not having a lab, we need a rapid diagnostic equipment…you know that kind of stuff, diagnosis is a problem, most time you may end up with a blind treatment when you don’t have a definitive result, you may end up with eh. you know a blind treatment that will or may not even yield anything, a patient comes to a doctor, you know, you must give a prescription to send away the patient and then move back in order…. in order for the lab to be ready, if you don’t give them anything, they will not be happy’. (IDI, Enugu, Pharmacy)"

### Reasons for not testing: community members views on accuracy of lab tests

Despite the nominal acceptance of testing as an important step towards appropriate treatment, there were obvious doubts in the outcomes of malaria tests; many respondents shared their personal experiences of the issue

"‘In the case of tests, sometimes, it records error for example; yesterday one of my brothers was having fever and after going for the test, the laboratory result recorded that there was nothing wrong with him. Later, we went to another laboratory and it came back positive with malaria parasite with typhoid and drugs were prescribed immediately but we’ve not still seen the result of the drug administered. But my concern is that if two laboratory results came back differently…what then is the need for testing?’ (P9, FGD, Adult Men)"

"‘There are some places you will go for lab test what this particular lab is saying is not what the other would say. The person ends up getting confused…. you understand?’ (P7, FGD, Udi, Primary caregiver)."

"‘I have not seen any difference because whenever I take my children to this lab they will be saying it is malaria parasite; every time malaria parasite, every time malaria parasite. When my neighbour will go malaria parasite, I don’t know if it is the same thing or not. Every time malaria parasite; all the labs malaria parasite; I don’t know’. (P4,FGD,Enugu, Adult Women)."

### Reasons for not testing: health provider’s perspective on accuracy of lab tests

The perceptions of providers of the accuracy of lab tests corroborate the responses from community members. Amongst providers, there was a general perception that test results are not always accurate which they attributed mainly to incompetencies of lab personnel.

"‘The microscopic result…they are trying but sometimes I think there are people that are not experts in that field, may be they may just handle it but due to carelessness they may mishandle some specimens that have been collected from another patient and this may have a contrary result’. (IDI Enugu, Health Centre, Nurse)."

"‘Sometimes one lab result might be different from another result of the same person, and same test is run’… The accuracy is not constant at all’. (IDI, Udi, Health Centre, Community Health Officer)"

### What people do with test results: community member’s reaction if test is negative

Among participants, there were expressions of suspicion and the need to go for another test for confirmation when initial test is a negative. There were other interpretations of negative test result:

"‘When a test comes out negative and the person is still sick, it may just be the handiwork of wicked people. The person may have been poisoned, but the doctor, if he is well grounded should refer the person to herbal treatment because negative means its poison and it will not appear on the test result’. (P4 FGD, Enugu, Adult Women)."

"‘I would want to know why they have not discovered what is wrong with me. I will go to another lab and do the test again. Because I know that am not well’. (P8 FGD, Udi, Adult Men)."

Some mothers expressed that they would continue treating with anti-malarials or get herbal medications for their children.

"‘If they say it is not malaria, I will get to my home town and get traditional medicine to take’. (P6 FGD, Enugu, Primary Caregivers)."

"‘I will not do anything. When I get home, I will continue to give the child anti-malarials’. (P3 FGD, Enugu, Primary Caregivers)."

### What people do with test results: Health providers’ views on peoples’ reaction to negative malaria tests

Health providers’ responses echo some of the responses provided by community participants. They reported that community members are generally not happy about negative test result, and often suspect the accuracy of such, while some others may think the sickness is due to supernatural forces.

"‘Some of them will be unhappy because if it is malaria, it is easier for them to treat but when the result is negative and you tell them to go to the hospital, their reaction is usually, what can this be? Some of them will by themselves suggest that they want to go for typhoid test. Surprisingly, a lot of patients do not want to go to hospital’. (IDI, Enugu, Pharmacy)"

"‘Most of them are even discouraged they don’t accept’ …they will be complaining saying you should try again …’ (IDI, Enugu, Health Centre, Nurse)"

"‘Some of the patients do take it seriously because in our context they always feel that everything is supernatural. If result is negative, it means that somebody must have bewitched them’. (IDI, Enugu, Health Centre, Nurse)"

### What people do with test result: testing is pointless if plan to give an antimalarial anyway

When asked about what they do with tests results, some health providers reported that malaria tests are not that important since in most instances treatment is based on what they see in a patient or what they are told.

"‘It is not particularly important; we can do without it that is when the symptoms are glaring’. (IDI, Enugu, pharmacy)."

Others mention that test results do not affect the type of treatment they give.

"‘……even if I did conduct any test maybe because of the signs and symptoms I have seen on the patients and I want to administer anti malaria treatment on the patient I will still use the same type of anti malaria’. (IDI, Enugu, Health centre, Nurse)"

## Discussion

This study shows that both providers and community members attach importance to the concept of testing especially to distinguish malaria from other illnesses and an important step towards appropriate treatment, however, what providers and community actually do with a test result is different. For instance some of the providers were of the opinion that test result does not affect the treatment they give. This finding is consistent with what has been reported elsewhere where despite the availability of diagnostics, patients with negative test results continued to receive anti-malarials [6,8]. This could be explained by the perception of providers that tests results are not always accurate due to their seeming lack of trust in the competence of laboratory personnel. Perhaps a more worrying aspect on the part of community members is the reported inconsistencies in test results leading them to question the need for tests. Such beliefs if not properly addressed could result in a lack of demand for tests even if they are made available, reinforce the current practice of presumptive treatment for febrile illness and in turn, lead to the over use of anti-malarials.

In this study, the reasons for not testing included the unavailability of testing equipment in health facilities. Despite the expressed desire to carry out tests, lack of testing equipment compels them to still use symptom-based approach for diagnosis; this could limit the ability of health providers to look for other causes of fever in non malaria febrile illnesses as all febrile cases are treated as malaria [8,18,19]. Thus it has been argued that providers should be equipped with testing facilities to enable treatment that is in line with a test result which will also safeguard the efficacy of ACT [20]. As reported in other setting [21], cost was mentioned as a barrier to testing and this is not surprising since individuals bear most of the cost of their treatment in this region and an additional cost of test may increase the burden of payment on patients and could result in their refusal to go for test. Though studies have identified that even when traditional barriers to testing such as availability and cost are minimized, people’s perceptions and practice behaviours emerge as continued barriers to use of test results [22].

An issue of concern emanating from this study is the notion of recommending tests to certain categories of people. In both the public and private facilities a common category was pregnant women, those who have previously had antimalarials but not recovered and patients with confusing symptoms. This could be because health messages have often portrayed certain categories of people like pregnant women as more vulnerable to malaria. While it suffices to give appropriate treatment to vulnerable groups, this impression could pose a problem if it becomes normal practice because even when these tests are made available, not everyone who presents with fever may get a chance to be tested as is currently being advocated. It will thus benefit National Malaria Control Programmes if health messages and treatment algorithms emphasize the importance of testing *every* febrile patient. Addressing this issue will help to improve access to proper treatment to everyone and to achieve the Roll Back Malaria (RBM) target of universal access to malaria diagnostic testing.

Both providers and community members expressed reservation and a lack of trust for a negative malaria test result and deem it necessary to repeat the same test for malaria elsewhere rather than explore other causes of the illness. This lack of trust in test result has been reported in other studies for microscopy as well as for RDTs [21,23,24] and have often resulted in continued prescription of antimalarials [8,24,25]. Other interpretations of negative test results were also found in this study, for instance there were views that in the event of a negative result, the illness could then be due to the activities of witchcraft or poison, or could be due to supernatural forces. This is similar to what was reported by Muela *et al*[26] where 62% of respondents shared the view that witches could interfere with normal malaria by hiding the parasites and making them invisible and thus undetectable. The authors attributed the interpretation of malaria in terms of witchcraft as being possible when biomedical treatment does not provide the expected outcome hence people give their own interpretation of the illness when the outcome does not conform to their preconception [26]. This belief could lead to further delays in seeking treatment and in treatment being sought from unorthodox health care providers.

One practical limitation of this study was that it was carried out at a time that RDTs had not been widely used by the cadre of health providers involved in the study, thus many people had not seen or experienced them. Their perceptions may thus differ when they have utilized these tests. Finally, this study was done in one state in the country and may not be generalisable to the whole country especially with existing diverse ethnic and religious groups.

In conclusion, the study shows that perceived importance of malaria tests by health providers and community members does not translate to trust in the results and thus test results may be of limited use in patient management. Consequently, introducing testing equipments may overcome the barrier of unavailability, but not some of the perceptions about accuracy and usefulness of tests. If tests are to be incorporated into treatment-seeking and provision, there should be behavioural change interventions for both the providers and community. In the first instance testing facilities should be made available in health facilities and health providers properly trained on how to perform tests. This should be backed up by supportive visits and supervision to ensure that providers adhere to testing guidelines and treat based on test results. Proper training has been shown to improve health provider’s ability to accurately perform tests, improve their confidence in the results and foster their acceptability in the community [14,27]. The use of supportive supervision was also reported to be effective elsewhere [19]. Health worker training on malaria case management should also emphasize better ways of communicating with patients on the need for tests. Treatment algorithms that clearly indicate to test everyone suspected of malaria should be provided, these algorithms should also indicate the course of action for a negative test result.

Though it is not evident on what mix of interventions will work in this setting, getting evidence about the effectiveness of alternative intervention strategies is important. A cluster randomized trial following on from this study is ongoing to assess the impact of a combination of approaches on treatment-seeking and provider prescribing in public and private facilities (clinicaltrials.gov NCT01350752).

## Competing interests

The authors declare that they have no competing interests.

## Authors’ contributions

CC, NE, OO, VW, LMJ & BU designed the study. NE, OE & LMJ were involved in data collection. NE & OE undertook data analysis; OE drafted the manuscript with assistance from all authors. OO, VW and BU provided guidance throughout the entire process. All authors read and approved the final manuscript.
